# Are paraspinous intramuscular injections of botulinum toxin a (BoNT-A) efficient in the treatment of chronic low-back pain? A randomised, double***-***blinded crossover trial

**DOI:** 10.1186/s12891-017-1816-6

**Published:** 2017-11-15

**Authors:** Mélanie Cogné, Hervé Petit, Alexandre Creuzé, Dominique Liguoro, Mathieu de Seze

**Affiliations:** 1 Service de Médecine Physique et de Réadaptation, hôpital Raymond Poincaré, 92380 Garches, France; 2 Service de Médecine Physique et de Réadaptation, CHU de Bordeaux, 33076 Bordeaux, France; 3 EA4136 Handicap, Activité, Cognition, Santé, Bordeaux University, Bordeaux, France; 4 Neurosurgical Unit, University Hospital, Bordeaux, France

**Keywords:** Low-back pain, Botulinum toxin, Disability, Function, Quality of life, Work

## Abstract

**Background:**

Treatment for patients with chronic low-back pain (LBP) is a public health issue. Intramuscular injections of botulinum toxin A (BoNT-A) have shown an analgesic effect on LBP in two previous randomized controlled studies. The objective of the study was to verify the efficacy of paravertebral injections of BoNT-A in patients with LBP.

**Methods:**

Patients were included in this phase 3 randomized double-blinded trial comparing the efficacy of BoNT-A versus placebo in a crossover study on LBP. Both groups received 200 units of BoNT-A in paravertebral muscles or a placebo, and vice versa at Day 120. The main judgment criterion was LBP intensity 1 month after the injections, evaluated by using a visual pain scale (VAS). Secondary assessment criteria included: LBP intensity 90 and 120 days after injection day; number of days when an allowed antalgic oral treatment was needed in between each evaluation; functional disability measured by the Quebec Back Pain Disability Scale; quality of life; inability to work; patient satisfaction in relation to the treatment’s effect; spinal mobility; and strength of spinal muscles, measured by isokinetic technique.

**Results:**

Nineteen patients completed the study. There was no significant difference between the groups’ average LBP during the last 8 days at Day30 (*p* = 0.97). There was no significant difference between the two groups regarding the secondary assessment criteria (*p* > 0.05).

**Conclusions:**

Injections of BoNT-A in the paravertebral muscles were not found to be effective to relieve chronic LBP. The limits of the study are that the dose of BoNT-A used was lower than in other studies, and that the limited number of patients included may explain the negative results.

**Trial registrations:**

Identifiers: NCT03181802. Unique Protocol ID: CHUBX2003.

## Background

Chronic low-back pain (LBP) is a public health problem that concerns 5 to 7% of the general occidental population [1] and has a significant impact on the quality of life of its sufferers [2].

Since the significance of lumbar stiffness in relation to contraction of the erector spinae muscles has been linked to the level of intensity of LBP [3], the lumbar erector spinae muscles have become a therapeutic target. Many recent arguments purport that paravertebral muscles have a predominant pathogenic role in perpetuating chronic back pain. During spinal movements, paravertebral muscles’ activity, recorded by electromyography, show abnormalities in subjects with low-back pain compared to subjects without LBP. A decrease in the power ratio between the erector spinae and flexor spinal muscles, measured by isokinetic techniques, is associated with chronic low-back pain. Finally, the significance of lumbar stiffness in relation to the erector spinae muscles contracting is linked to the level of intensity of low-back pain [3]. Local muscular treatments have already been tried such as physiotherapy, massage, infrared therapy and botulinum toxin A (BoNT-A) [4–6].

In addition to its muscle-relaxing effect, local intramuscular injections of BoNT-A have also shown an analgesic effect on pain related to dystonia, tension headaches, myofascial pain syndrome and chronic neck pain [7–11]. This effect is usually reversible after 3 months. Foster et al. [4] used BoNT-A A for its peripheral muscle relaxant action as a local intramuscular treatment of chronic LBP. This double-blinded, placebo, controlled trial in 31 patients showed that paravertebral administration of BoNT-A in patients with chronic LBP relieved pain and improved function at 3 and 8 weeks after treatment. Machado et al. [6] showed also in a randomized controlled trial that BoNT-A injections relieved pain and improved quality of life of 19 patients at 4 weeks. Further open studies have been performed to value the efficacy of BoNT-A in patients with chronic LBP [12–15] but all of them aimed to establish predictive factors of pain relief, and the efficacy was limited to 3 months. A Cochrane meta-analysis [16] concluded that “there was low quality evidence in the short term, and very low quality in the intermediate term, that BoNT-A injections reduced pain intensity more effectively than saline injections in participants with LBP” and that “there was very low quality evidence that BoNT-A injections compared to corticosteroid injections could reduce chronic LBP intensity in the short term”.

Studying the therapeutic effect of paravertebral injections of BoNT-A requires further studies to confirm the reported short-term therapeutic effect and to determine potential predictive factors of efficacy.

### Objectives of this trial

Main objective: To evaluate the analgesic effect 1 month after a single injection of 200 IU of BoNT-A in 10 bilateral paravertebral intramuscular points for treating chronic LBP.

### Secondary objectives

- To evaluate the analgesic effect of paravertebral injections of BoNT-A 3 months after its administration in chronic LBP sufferers.
- To measure the impact of paravertebral injections of 200 IU of BoNT-A in a single administration on lumbar stiffness and on spinal extensor muscle strength in patients with chronic LBP.
- To search for predictive factors of the analgesic effect of BoNT-A injections.

## Materials and methods

This study was a randomized**,** double**-**blinded**,** placebo-controlled phase 3 trial comparing BoNT-A Type A injections (Botox) to a placebo in patients with chronic LBP (Level 2, OCEBM Levels of Evidence Working Group*, "The Oxford 2011 Levels of Evidence"). This superiority trial obtained support from the French Hospital Clinical Research Project (PHRC).

The number of participants included in the study was similar as those included in the previous study (see [4]), that showed a strong positive effect of BoNT-A injections on LBP. Furthermore, the design of our study (i.e. a crossover) increased the power of the statistical analysis. In this context, 60 inclusions were planned (30 in each group). Nevertheless, regular intermediary analyses were planned by an independent scientific committee, to ensure that the trial did not present any secondary effect, or that we could conclude in an intermediary step that BoNT-A was inefficient in pain relief. After obtaining a similar number of injections than Foster, the study was stopped by the scientific committee, because there was no trend in pain relief.

### Ethics, consent and permissions

This trial obtained the approval of a French ethics Committee (2003/02) and all participants received an information note and gave their written informed consent. The clinical trial registration number was: Identifiers: NCT03181802, Unique Protocol ID: CHUBX2003.

### Population

The patients included were consulted by Physical and Rehabilitation Medicine spinal pathology specialists at the University Hospital of Bordeaux, met the eligibility criteria, and volunteered to participate in the study.

Inclusion criteria were: LBP defined as a pain located between the thoracic lumbar hinge and the gluteal sulcus, where pain had evolved over a period of 6 months despite well conducted medical treatment, self-assessed lumbar pain intensity over 50 mm long on a visual analogue scale of 100 mm (0 = no pain; 100 = maximal pain), having been on sick leave for 60 or more days in the year preceding the inclusion (in order to include patients with high consequences of chronic low-back pain on their work), the same long-term chronic pain treatment for at least 6 weeks, and a paravertebral painful point pressure.

Exclusion criteria were: age under 18 or over 55 years (to avoid secondary causes of low back pain, like spinal tumor), ongoing pregnancy or breast-feeding, a neuromuscular pathology (myasthenia gravis, amyotrophic lateral sclerosis, myopathy, polymyositis), aminoglycoside treatment at the time of inclusion, skin infection at injection points, diabetes and alcoholism (in order to avoid other etiologies of chronic pain), a history of injecting BoNT-A A, anticoagulation treatment, sciatica, suspected spinal inflammatory disorder (spondylitis, inflammatory rheumatism, tumoral pathology), a failed back surgery syndrome (when surgery failed to relieve low-back pain), incapacity to stand, cardiorespiratory deficiency which does not allow the isokinetic exploration of the spinal muscles, cognitive disorders limiting patient participation, conflicts of interest owing to existing pain (unconsolidated work accident, ongoing damage compensation). Spine infection, tumour or trauma had been specifically excluded by an MRI done by all patients before the inclusion in the present study. Some of risk factors associated with going from acute low-back pain to chronic low-back pain are linked to the socio-professional context, notably with the job dissatisfaction [17, 18]. Furthermore, 2 studies [17, 19] showed that there was a significant positive association between a damage compensation and chronic incapacity. In general, patients with unconsolidated work accident or ongoing damage compensation have a higher probability to be at risk of chronic disease; they also have a lower probability to positive response to treatment in general. That is why we excluded them from the study. We measured it by asking to each participant: “are you currently in an unconsolidated work accident?” and “are you currently ongoing a damage compensation?”. As a High Authority of Health in France (l’Agence Nationale d’Accréditation et d’Evaluation en Santé, Diagnostic, Prise en charge et suivi des malades atteints de lombalgie chronique, Décembre 2000) classified the beginning of a LBP after the age of 55 as an « alert sign », we excluded them from the study.

No patient was allowed to take opiates during the time of the study, and facet joint injections were also not permitted during the study period. Physiotherapy programs offered during the study period were isometric exercises and core muscle strengthening exercises one or twice per week (usual physiotherapy in chronic low-back pain, that patients made before the study, and which was not modified during the study).

### Experimental procedure

#### Task

The design of this study was a crossover. The subjects were randomized into two groups and successively received the two treatments of the study: patients in group 1 received intramuscular paravertebral injections of BoNT-A during the first sequence of treatment, then a placebo during the second sequence of treatment 120 days later; patients in group 2 received a placebo during the first sequence of treatment, then intramuscular paravertebral injections of BoNT-A during of the second sequence of treatment 120 days later. The crossover was performed 120 days after the inclusion in the study, because most patients with initial improvement induced by BoNT-A injections reported in previous studies [12] that the beneficial effect waned at four months.

A paper table of randomization was used by the pharmacist at the University Hospital of Bordeaux (block randomization with block size of 6). The pharmacist who performed the randomization was blinded to the patient’s characteristics.

Therapeutic procedure: For each group, the injected solution was prepared by the hospital pharmacist in order that both the patients and the injectors were blinded to the nature of the injected solution. The treatments compared were: 200 IU of BoNT-A diluted in 4 ml of physiological saline injected intramuscularly in the paravertebral lumbar muscles, versus 4 ml of physiological saline injected intramuscularly in the paravertebral lumbar muscles (placebo). The injector administered the solution in 10 intramuscular puncture points (0.4 mL/point) equally distributed from L1 to L5, bilaterally. The site of injection was detected by electromyography using the injection needle. No complementary pain treatment was prescribed after the injections.

Follow-up: patients were examined at inclusion Days 0, 30, 90, 120 (Day of the crossover), 150, 210 and 240, i.e., D0, D30, D90 and D120 after both sessions of injection. The follow-up was done in person. Patients were blinded throughout the entire study.

#### Measures

The main judgment criterion was the level of LBP intensity at D30 (when the maximal effect of BoNT-A injections is anticipated). Pain intensity was measured on a horizontal visual analogue scale 100 mm long, with « no pain » written on one end and « maximum pain » on the other (0 = no pain; 100 = maximal pain). The question asked was: “How was the intensity of your LBP over the last 8 days?” To consider the pain decrease as clinically significant, we used the guidelines of Pham et al. [20], who suggested that a change of 40 mm could be clinically significant.

### Secondary judgment criteria

- Initial pain was detailed as follows: Immediate average LBP was recorded on VAS at the first injection (D0). Average pain intensity over the last week and the last month were also recorded at D0, with the same horizontal visual analogue scale.
- Lumbar pain intensity at D90 and D120 was measured on a horizontal visual analogue scale 100 mm long, with « no pain » written on one end and “maximum pain” on the other. The question asked was: “How was the intensity of your LBP over the last 8 days?” (0 = no pain; 100 = maximal pain).
- The number of days when oral pain treatment (antalgic or non-steroid anti-inflammatory, opiates were not permitted) between evaluation times was taken. Days when treatment was taken were noted as they occurred by the patient in a calendar, which was distributed at D0. We thought that a change of 25% would be significant.
- Functional disability related to LBP was measured by the Quebec Back Pain Disability Scale at each evaluation time. The higher the score (/100), the higher the disability. We considered as determined by Ostelo et al. [21] 20 points of change of the Quebec score as clinically significant.
- Quality of life was measured at each evaluation time on a horizontal visual analogue scale 100 mm long. The question asked was: "In your opinion, how was your quality of life over the last month?" (0 = no impact to 100 = major deterioration). We considered as clinically significant a change of 0.2 standard deviation (small change), 0.5 standard deviation (moderate change) and 0.8 standard deviation (large change) [22, 23].
- Inability to work was measured by a compendium of data indicating the number of sick leave days due to LBP in the 8 months preceding inclusion and during follow-up. A change of 25% was considered as clinically significant.
- Patient satisfaction regarding the effect of the treatment was measured on a horizontal visual analogue scale 100 mm long at each evaluation time. The question asked at each evaluation was: "In your opinion, how is the overall efficacy of the treatment that you have received?"(0 = no efficacy; 100 = high efficacy). A change superior than 50% was considered as clinically significant.
- Spinal mobility was measured at each evaluation time by using Schober & Macrae’s test (Miller 1984). Two lines were drawn 10 cm above the postero superior iliac spine and 5 cm below the postero superior iliac spine. The distances in a standing position and in anteflexion were measured. A difference less than 4 cm was considered as a spine stiffness.
- Spinal muscle strength was measured by flexion and extension isokinetic technique at a speed of 60° per second before the injections, at D30, D120, D150 and D240. A variation of strength up to 20% or a reversal of the flexor/extensor ratio was considered as clinically significant.
- MODIC classification of discopathy and Hadar classification of the rector spinae muscles were based on MRI performed in the previous year. The MODIC measures are divided in 3 classes: [24]: there were type 1 (inflammatory phase), type 2 (fatty phase) and type 3 (marked sclerosis adjacent to the endplates). We collected the data in order to look for predictive factors for efficacy of BoNT-A.
- Tolerance to BoNT-A injections was studied by actively asking at each visit for possible side effects (pain at injection points, sensation of general weakness, falling, nausea, diplopia, dry mouth).

### Statistical analysis

Comparisons were made by a paired Student t-test after verifying the conditions of validity of the test (normal distribution, homogeneous variances). The Chi square test was used in order to compare the gender distribution of the two groups. Paired t-tests and Chi square tests were performed on cumulative data from 19 patients following placebo (19 patients) and BoNT-A injections (19 patients) after a crossover. Linear regression analysis was also planned. Risk of type 1 error was α = 5% at each statistical analysis. To run the statistical analyses, we used the Excel software, version 15.32. The statistician who decided the kind of statistics used was blinded. The author who made the statistical analyses was not blinded, but he/she did not compile the data into the statistical software.

## Results

The group who began the injections with BoNT-A was named group 1; the group who began the injections with a placebo was named group 2. As planned, in order to increase the power of the statistical analysis of the crossover, we pooled post-BoNT-A follow-up and post-placebo follow-up. The group with BoNT-A injections was named group A and the group with placebo injections was named group B. The follow-up of groups A and B was performed at D30, D90 and D120 following each injection time.

### Flow diagram (Figure 1)

**Fig. 1:**
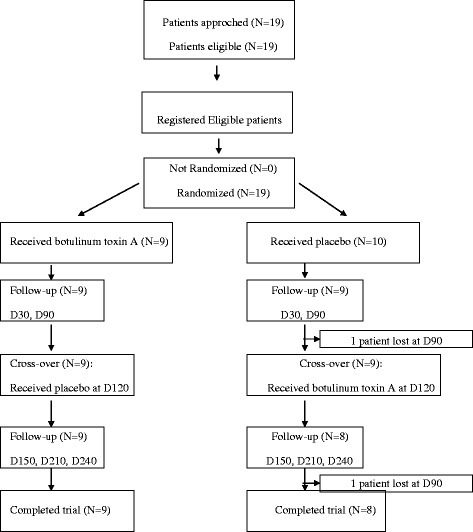
Flow diagram

In this study, 19 patients were approached and eligible to the study. No patient declined participation in the study. The inclusion period was about 23 months. All patients included were randomized in one of the two groups. Nine of them received BoNT-A at D0, 10 of them received placebo at D0. In the BoNT-A group (group 1), all patients were followed at D30, D90, all of them received placebo at D120, were followed at D150, D210 and D240 and completed the trial. In the placebo group (group 2), one patient was lost during the follow-up at D90 and one patient was lost during the follow-up at D210; 8 patients received BoNT-A at D120, all of them were followed at D150, D210 and D240 and completed the trial. We excluded the 2 lost patients from the statistical analysis, because they did not benefit from the 2 injections (BoNT-A and placebo). Patients' distribution is presented in Fig. 1.

### Description of the population at baseline (standard deviations are noted in parentheses) (Table 1)

The group who initially received BoNT-A was named group 1; the group who initially received the placebo was named group 2. The group 1 contained 6 women and 3 men, and the group 2 contained 10 women (Chi square = 3.96, *p* = 0.047). There was no significant difference concerning the mean age of group 1 (38.1(±5.94)) and group 2 (38.2(±10.27)) (*p* = 0.98). The mean usual spinal pain intensity of group 1 was 59.33 mm (±15.71) and the one of group 2 was 58.70 (±15.89) (*p* = 0.93). The usual root pain intensity did not differ between groups either (*p* = 0.26) (mean pain intensity in group 1: 42.89 mm (±26.98); mean pain intensity in group 2: 28.40 mm (±27.16)). The mean pain intensity during the last month was 63.11 mm (±25.70) in group 1 and 66.70 mm (±24.50) in group 2 (*p* = 0.76); the mean pain intensity during the last 8 days was 67.67 mm (±22.37) in group 1 and 57.50 mm (±25.63) in group 2 (*p* = 0.37). There was no significant difference concerning the mean Quebec initial score between group 1 (52.56 mm (±11.64)) and group 2 (51.70 mm (±16.55)) (*p* = 0.90). There was no significant difference concerning the mean disability during the last month between group 1 (7.44 mm (±12.99)) and group 2 (13.4 mm (±14.55)) (*p* = 0.36); but the disability during the last 8 months was higher in the group 2 (151.6 mm (±96.56)) than in the group 1 (58.22 mm (±82.29)) (*p* = 0.03). The quality of life at inclusion was estimated at 76.56 mm (±16.41) for group 1 and at 65.00 mm (±17.80) for group 2 (*p* = 0.16). There was no significant difference concerning the number of days with painkillers or anti-inflammatories between group 1 (19.67 days (±13.44)) and group 2 (14.1 days (±12.57)) (*p* = 0.36). In group 1, 4 patients had a right-, 3 had a left- and 2 had a bilateral paravertebral painful point pressure; in group 2, 4 patients had a right- and 6 patients had a bilateral paravertebral painful point pressure. In group 1, 8 patients and 6 patients of group 2 had a stiffness (*p* = 0.17). The Schober’s test was measured at 4.22 cm (±1.30) for group 1 and 3.95 cm (±1.77) for group 2 (*p* = 0.71). The hand-ground distance was about 28.60 cm (±13.60) in group 1 and 20.60 (±15.60) for group 2 (*p* = 0.25). The mean number of localization of spinal pain was 3.13 (±1.46) in group 1 and 3.60 (±1.84) in group 2 (*p* = 0.55), and the mean number of localization of paravertebral pain was 5.00 (±1.51) in group 1 and 4.60 (±2.32) in group 2 (*p* = 0.67). No patients presented a Lasegue sign at the inclusion; 2 patients presented a pseudo-Lasegue sign in group 1 and 5 presented a pseudo-Lasegue sign in group 2 (*p* = 0.30). Only one patient in group 2 presented a disco-radicular conflict (*p* = 0.34). The isokinetic evaluation revealed a maximum strength at 115.33n/m (±58.63) in group 1 and at 114.44n/m (±37.63) in group 2 (*p* = 0.97); the endurance was calculated at 89.11n/m (±62.50) in group 1 and 77.33n/m (±53.86) in group 2 (*p* = 0.67); the flexors/extensors ratio at 60° was calculated at 123.63% (±37.56) in group 1 and 119.36 (±49.51) in group 2 (*p* = 0.84). Population at baseline is described in Table 1.

**Table 1:** Demographic data of 19 randomized patients (mean or number are noted, standard deviations are in parentheses) at Day 0 (D0)

Patients	Botulinum toxin	Placebo	t-test (p)
Sample size	*N* = 9	*N* = 10	
Men/Women	3/6	0/10	0.047
Age: mean (SD) in years	38.1 (5.94)	38.2 (10.27)	0.49
Spinal pain intensity: mean (SD) /100 mm	59.33 (15.71)	58.70 (15.89)	0.47
Radicular pain intensity: mean (SD) /100 mm	42.89 (26.98)	28.40 (27.16)	0.13
Pain intensity during last month: mean (SD) /100 mm	63.11 (25.70)	66.70 (24.50)	0.38
Pain intensity during last week: mean (SD) /100 mm	67.67 (22.37)	57.50 (25.63)	0.18
Quebec initial score mean (SD) /100 mm	52.56 (11.64)	51.70 (16.55)	0.45
Disability during last 8 months: mean (SD) /100 mm	58.22 (82.29)	151.6 (96.56)	0.018
Disability during last month: mean (SD) /100 mm	7.44 (12.99)	13.4 (14.55)	0.18
Quality of life at inclusion: mean (SD) /100 mm	76.56 (16.41)	65.00 (17.80)	0.08
Number of days with painkillers or anti-inflammatories: number (SD)	19.67 (13.44)	14.1 (12.57)	0.18
Paravertebral painful point pressure Right/Left/Bilateral	4/3/2	4/0/6	
Stiffness: number	8	6	0.08
Tendency to cough: number	5	7	0.27
Instability: number	9	8	0.08
Schober’s test: centimeter (SD)	4.22 (1.30)	3.95 (1.77)	0.35
Hand-ground distance: centimeter (SD)	28.60 (13.60)	20.60 (15.60)	0.13
Spinal pain: mean (SD)	3.13 (1.46)	3.60 (1.84)	0.27
Paravertebral pain: number	2	5	0.33
Lasegue sign: number	0	0	
Pseudo-Lasegue sign: mean (SD)	0.25 (0.46)	0.5 (0.53)	0.15
Disco-radicular conflict: number	0	1	0.17
MODIC L1-L2 0/1/2/3	9/0/0/0	9/0/1/0	0.17
MODIC L2-L3 0/1/2/3	9/0/0/0	9/0/1/0	0.17
MODIC L3-L4 0/1/2/3	9/0/0/0	9/0/1/0	0.17
MODIC L4-L5 0/1/2/3	9/0/0/0	9/0/0/1	0.17
MODIC L5-S1 0/1/2/3	5/3/1/0	4/3/2/1	0.15
HADAR L1-L2 0/1/2/3	8/1/0/0	8/2/0/0	0.31
HADAR L2-L3 0/1/2/3	6/3/0/0	7/3/0/0	0.44
HADAR L3-L4 0/1/2/3	4/5/0/0	7/1/2/0	0.33
HADAR L4-L5 0/1/2/3	1/6/2/0	3/4/3/0	0.37
HADAR L5-S1 0/1/2/3	1/4/4/0	0/4/6/0	0.18
Isokinetic maximum strength: n/m (SD)	115.33 (58.63)	114.44 (37.63)	0.49
Isokinetic endurance: n/m (SD)	89.11 (62.50)	77.33 (53.86)	0.34
Flexors/extensors ratio at 60°: % (SD)	123.63 (37.56)	119.36 (49.51)	0.42

### Between-group comparisons (Table 2)

#### Level of LBP intensity

Between-group comparisons are presented in Table 2.

**Fig. 2:**
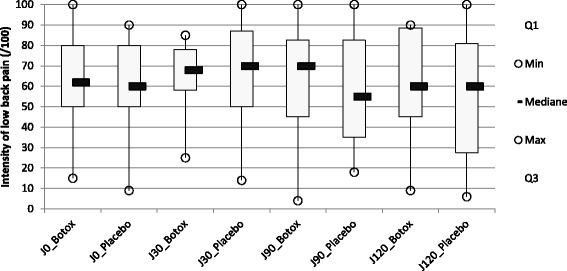
Pain intensity at D0,30, 90 and 120 for patients treated by Botulinum toxin A (BoNT-A) (group A) or by placebo (group B). Pain intensity was measured on a horizontal visual analogue scale 100 mm long, with « no pain » written on one end and « maximum pain » on the other. The question asked was: “How was the intensity of your low-back pain over the last 8 days?”

- LBP intensity during the last 8 days ( Fig. 2 ):

**Table 2:** Presentation of averages, standard deviations and *p*-values of judgment criteria for group A and group B

		Number of patients (n) A/B	Mean group A	Standard deviation group A	Mean group B	Standard deviation group B	*p*-value
Average lumbar pain over last 8 days by visual analogue scale (/100 mm)	*D0*	18/19	67.70	24.64	60.35	28.07	*p* = 0.43
	*D30*	18/19	63.12	18.92	63.12	18.92	*p* = 0.75
	*D90*	15/16	62.60	27.39	58.43	24.66	*p* = 0.80
	*D120*	15/16	60.87	26.83	55.87	32.50	*p* = 0.70
Average root pain over last month by visual analogue scale (/100 mm)	*D30*	18/19	60.29	22.99	53.47	33.88	*p* = 0.45
	*D90*	15/16	42.07	37.40	27.57	33.05	*p* = 0.52
	*D120*	15/16	56.73	25.33	46.20	30.42	*p* = 0.70
Number of days with significant or very significant pain	*D30*	18/19	13.29	9.88	15.18	12.82	*p* = 0.55
	*D90*	15/16	11.43	10.45	11.71	16.94	*p* = 0.44
Functional disability related to low-back pain by Quebec Back Pain Disability Scale (/100)	*D0*	18/19	51.53	16.19	52.35	20.16	*p* = 0.89
	*D30*	18/19	53.76	13.18	52.29	20.74	*p* = 0.77
	*D90*	15/16	53.07	17.75	45.93	22.82	*p* = 0.47
	*D120*	16/16	52.87	21.69	42.93	23.70	*p* = 0.48
Inability to work during last 30 days (/30)	*D0*	18/19	11.00	14.56	11.06	14.55	*p* = 0.99
	*D30*	18/18	12.41	15.17	9.56	14.25	*p* = 0.35
	*D90*	15/16	12.41	14.59	9.69	17.93	*p* = 0.46
	*D120*	15/16	8.00	13.73	12.00	15.21	*p* = 0.34
Estimated impact of low-back pain on quality of life (/100)	*D0*	18/19	71.41	21.70	64.47	24.61	*p* = 0.37
	*D30*	18/19	68.71	18.85	64.06	23.33	*p* = 0.44
	*D90*	15/16	63.47	23.72	58.57	23.33	*p* = 0.38
	*D120*	15/16	61.00	30.01	60.67	29.63	*p* = 0.96
Number of days when pain medication or anti-inflammatories were necessary in last 30 days (/30)	*D0*	18/19	17.06	13.65	15.35	14.69	*p* = 0.71
	*D30*	18/19	16.06	13.21	13.41	13.44	*p* = 0.51
	*D90*	15/16	15.73	13.85	11.50	13.82	*p* = 0.79
	*D120*	15/16	14.80	14.87	13.53	14.54	*p* = 0.86
Patients’ assessment of efficacy of treatment (/100)	*D30*	18/19	0.76	1.15	0.94	1.14	*p* = 0.62
	*D90*	15/16	1.33	1.80	1.43	1.60	*p* = 1.00
	*D120*	15/16	1.47	1.77	1.73	1.62	*p* = 1.00
Spinal flexibility measured by Schoeber Macrae’s test (cm)	*D0*	18/19	5.00	2.34	4.21	1.86	*p* = 0.22
	*D30*	18/19	4.76	1.88	4.32	1.67	*p* = 0.48
	*D90*	15/16	4.00	1.18	4.54	1.31	*p* = 0.23
	*D120*	15/16	4.23	1.55	5.53	2.28	*p* = 0.18
Hand-ground distance (cm)	*D0*	18/19	26.17	13.32	25.64	14.03	*p* = 0.93
	*D30*	18/19	26.35	14.02	26.85	12.89	*p* = 0.92
	*D90*	15/16	24.87	14.51	16.79	10.17	*p* = 0.35
	*D120*	15/16	27.53	12.18	21.93	13.08	*p* = 0.25
Isokinetic maximum strength (n/m)	*D0*	16/17	116.00	45.53	126.40	63.41	*p* = 0.78
	*D30*	15/18	120.93	53.30	134.40	63.67	*p* = 0.70
	*D120*	13/14	126.69	67.20	135.07	50.35	*p* = 0.70
Isokinetic endurance (n/m)	*D0*	16/17	100.27	51.63	102.33	63.24	*p* = 0.81
	*D30*	15/17	96.07	53.38	108.73	69.00	*p* = 0.76
	*D120*	15/14	103.17	63.74	111.79	57.67	*p* = 0.65
Isokinetic maximum force ratio flexors/extensors (%)	*D0*	16/17	115.85	31.13	119.72	38.95	*p* = 0.36
	*D30*	15/17	122.93	33.83	107.86	24.41	*p* = 0.16
	*D120*	13/14	111.54	24.29	102.88	23.53	*p* = 0.72

Group A: all 17 patients assessed during 120 days after BoNT-A injections, group B: all 17 patients assessed during 120 days after placebo injections

There was no significant difference concerning the mean of the LBP intensity during the last 8 days between group 1 and group 2 at D30 (*p* = 0.59), at D90 (*p* = 0.94), at D120 (*p* = 0.73), at D150 (*p* = 0.92), at D210 (*p* = 0.80) and at D240 (*p* = 0.36).

There was no significant difference concerning the mean of the LBP intensity during the last 8 days between group A and group B at D30 (*p* = 0.75), at D90 (*p* = 0.80) and at D120 (*p* = 0.70).

- Root pain intensity over the last month:

There was no significant difference concerning the mean root pain over last month between group 1 and group 2 at D30 (*p* = 0.31), at D90 (*p* = 0.23), at D120 (*p* = 0.54), at D150 (*p* = 0.92), at D210 (*p* = 0.77) and at D240 (*p* = 0.46).

There was no significant difference concerning the mean root pain over last month between group A and group B at D30 (*p* = 0.45), at D90 (*p* = 0.51) and at D120 (*p* = 0.70).

- Number of days with significant or very significant pain:

There was no significant difference concerning the number of days with significant or very significant pain between group 1 and group 2 at D30 (*p* = 0.63), at D90 (*p* = 0.94), at D120 (*p* = 0.94), at D150 (*p* = 0.27), at D210 (*p* = 0.68) and at D240 (*p* = 0.64).

There was no significant difference concerning the number of days with significant or very significant pain between group A and group B at D30 (*p* = 0.55), at D90 (*p* = 0.44) and at D120 (*p* = 0.35).

#### Functional disability related to LBP evaluated by Quebec back pain disability scale (figure 3)

There was no significant difference concerning the score of the Quebec scale between group 1 and group 2 at D30 (*p* = 0.86), at D90 (*p* = 0.89), at D120 (*p* = 0.94), at D150 (*p* = 0.65), at D210 (*p* = 0.35) and at D240 (*p* = 0.13).

**Fig. 3:**
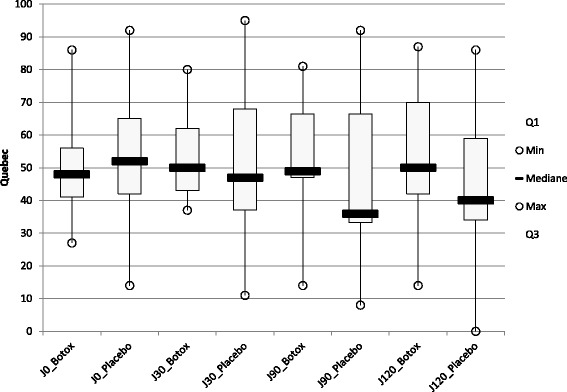
Quebec Back Pain Disability Scale at D0, 30, 90 and 120 for patients treated by Botulinum toxin A (group A) or by placebo (group B)

There was no significant difference concerning the score of the Quebec scale between group A and group B at D30 (*p* = 0.77), at D90 (*p* = 0.47) and at D120 (*p* = 0.48).

#### Inability to work during the last 30 days

There was no significant difference concerning the number of days with inability to work during the last 30 days between group 1 and group 2 at D30 (*p* = 0.35), at D90 (*p* = 0.46), at D120 (*p* = 0.27), at D150 (*p* = 0.10), at D210 (*p* = 0.47) and at D240 (*p* = 0.86).

There was no significant difference concerning the number of days with inability to work during the last 30 days between group A and group B at D30 (*p* = 0.35), at D90 (*p* = 0.46) and at D120 (*p* = 0.34).

#### Estimated impact of LBP on quality of life (figure 4)

There was no significant difference concerning the estimating impact of LBP on quality of life during the last month between group 1 and group 2 at D30 (*p* = 0.38), at D90 (*p* = 0.56), at D120 (*p* = 0.90), at D150 (*p* = 0.98), at D210 (*p* = 0.98) and at D240 (*p* = 0.93).

**Fig. 4:**
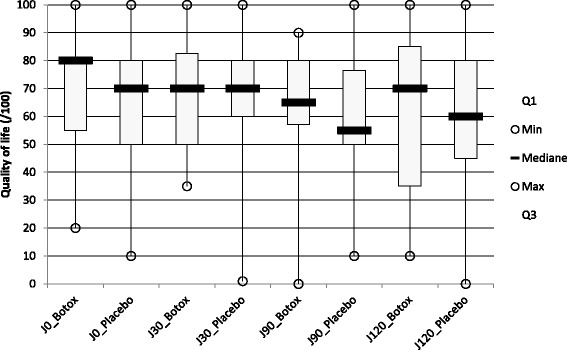
Estimated impact of low-back pain on quality of life at D0, D30, D90 and D120 for patients treated by Botulinum toxin A (group A) or by placebo (group B). It was measured on a horizontal visual analogue scale 100 mm long. The question asked was: "in your opinion, how was your quality of life over the last month?" (0 = no impact to 100 = major deterioration)

There was no significant difference concerning the estimating impact of LBP on quality of life during the last month between group A and group B at D30 (*p* = 0.44), at D90 (*p* = 0.38) and at D120 (*p* = 0.95).

#### Number of days when pain medication or anti-inflammatories were necessary in last 30 days

There was no significant difference concerning the number of days when pain medication or anti-inflammatories were necessary in the last 30 days between group 1 and group 2 at D30 (*p* = 0.82), at D90 (*p* = 0.51), at D120 (*p* = 0.73), at D150 (*p* = 0.57), at D210 (*p* = 0.58) and at D240 (*p* = 0.92).

There was no significant difference concerning the number of days when pain medication or anti-inflammatories were necessary in the last 30 days between group A and group B at D30 (*p* = 0.51), at D90 (*p* = 0.79) and at D120 (*p* = 0.86).

#### Patients’ assessment of efficacy of treatment

There was no significant difference concerning the patients’ assessment of efficacy of treatment between group 1 and group 2 at D30 (*p* = 0.73), at D90 (*p* = 0.69), at D120 (*p* = 0.89), at D150 (*p* = 0.91), at D210 (*p* = 0.64) and at D240 (*p* = 0.51).

There was no significant difference concerning the patients’ assessment of efficacy of treatment between group A and group B at D30 (*p* = 0.62), at D90 (*p* = 1.00) and at D120 (*p* = 1.00).

#### Spinal flexibility measured by Schoeber Macrae’s test

There was no significant difference concerning the spinal flexibility measured by Schoeber Macrae’s test between group 1 and group 2 at D30 (*p* = 0.49), at D90 (*p* = 0.06), at D120 (*p* = 0.30), at D150 (*p* = 0.64), at D210 (*p* = 0.47). There was a significant difference between group 1 and group 2 concerning the spinal flexibility at D240 (*p* = 0.04).

There was no significant difference concerning the spinal flexibility measured by Schoeber Macrae’s test between group A and group B at D30 (*p* = 0.48), at D90 (*p* = 0.23) and at D120 (*p* = 0.18).

#### Hand-ground distance

There was no significant difference concerning the hand-ground distance between group 1 and group 2 at D30 (*p* = 0.64), at D90 (*p* = 0.10), at D120 (*p* = 0.33), at D150 (*p* = 0.41) and at D210 (*p* = 0.81). There was a significant difference concerning the hand-ground distance between group 1 and group 2 at D240 (*p* = 0.58).

There was no significant difference concerning the hand-ground distance between group A and group B at D30 (*p* = 0.92), at D90 (*p* = 35) and at D120 (*p* = 0.25).

#### Isokinetic maximum strength

There was no significant difference concerning the isokinetic maximum strength between group 1 and group 2 at D30 (*p* = 0.34), at D120 (*p* = 0.30) and at D150 (*p* = 0.11). There was a significant difference concerning the isokinetic maximum strength between group 1 and group 2 at D240 (*p* = 0.04).

There was no significant difference concerning the isokinetic maximum strength between group A and group B at D30 (*p* = 0.70) and at D120 (*p* = 0.70).

#### Isokinetic endurance

There was no significant difference concerning the isokinetic endurance between group 1 and group 2 at D30 (*p* = 0.26), at D120 (*p* = 0.21) and at D150 (0.08). There was a significant difference between group 1 and group 2 concerning the isokinetic endurance between group 1 and group 2 at D240 (*p* = 0.03).

There was no significant difference concerning the isokinetic endurance between group A and group B at D30 (*p* = 0.76) and at D120 (*p* = 0.65).

#### Isokinetic maximum force ratio flexors/extensors

There was no significant difference concerning the isokinetic maximum force measured by the radio flexors/extensors at 60° between group 1 and group 2 at D30 (*p* = 0.90), at D120 (0.89), at D150 (0.08), at D240 (0.19).

There was no significant difference concerning the isokinetic maximum force measured by the radio flexors/extensors at 60° between group A and group B at D30 (*p* = 0.16) and at D120 (*p* = 0.72).

### Within-group comparisons

There was no significant difference in group A and in group B concerning the pain intensity between D0 and D120 (*p* = 0.58 for group A and *p* = 0.70 for group B).

### Symmetric carryover effect

There was a symmetric carryover effect between group 1 and group 2 concerning the main judgement criterion, i.e. pain intensity at D30.

### Adverse effects

The adverse effects were actively asked at each visit. No patients declared an adverse effect during the present study. No complications were experienced in this study.

## Discussion

This randomized controlled trial did not find any advantage for injections of BoNT-A versus placebo in the paravertebral muscles of patients with LBP at 30, 90 et 120 days with regard to pain relief, functional disability, sick leave, quality of life, consumption of oral antalgics, spinal flexibility and isokinetic strength or endurance. Indeed, there was no significant difference between the two groups regarding the main criterion, i.e.*,* average lumbar pain over the last 8 days at D30 (*p* = 0.97), nor was there any significant difference between the two groups regarding secondary judgment criteria (*p* > 0.05, see Table 2).

Our results differ largely from those of two previous studies [4, 6]. Since LBP is a complex phenomenon involving heavy lifting, twisting and trauma which is sometimes work-related [25] psychological factors [26], smoking, alcoholism, biomechanical and psychosocial professional factors, the difference in results could be due to differences in the populations of the two studies. Indeed, in a previous study [4], 3 patients had a discectomy compared to none in our study. In addition, no patient had any MRI evidence of acute disc pathology in the two previous studies [4, 6], whereas 6 patients in our study had a MODIC 1. Furthermore, in our study, only 3 male patients were included. Nevertheless, there was no difference between groups concerning the gender. Furthermore, the present literature is not uniform about the role of the gender on the chronicity of low-back pain [27, 28].

In our study, we used 200 units of Botox for bilateral injections. But we assume that the negative results of our study could also be secondary to the lower dose of botulinum toxin A used on each injection point compared to Foster et al.’s study. Indeed, we decided to inject bilaterally the paravertebral muscles, because there is usually a bilateral injury in both paravertebral muscles after an acute low back-pain, which leads to chronic low-back pain [29]. More precisely, we think that LBP could be secondary to an over-activity of muscles compensating multifidus’ atrophy. The dose used in Machado et al.’s study (1000UI of Dysport in case of bilateral injections) was also superior to ours, which could explain the difference between the results.

Reporting a negative study is still an interesting point, because the efficiency of BoNT-A on chronic low-back pain is still not proved at this time, and because it could make reconsider on one part in researchers’ further studies about BoNT-A and LBP, and one the other part in clinicians using BoNT-A injections for chronic LBP.

The strength of this trial is its randomized, controlled crossover design. A limitation is the small sample. Nevertheless, the number of patients treated was similar to that in previous studies [4, 6, 14], which showed a strong positive effect of BoNT-A on LBP. While some differences between the groups became apparent before the crossover (hand-ground distance, Schober’s test, isokinetic measures), they disappeared when the data were aggregated after the crossover (group A and group B), perhaps owing to variations due to the size of groups 1 and 2. Indeed, group 1 demonstrated more spine stiffness and less strength than group 2, a finding that was unexpected.

The main result of the study is the absence of any significant difference or trend to feel pain relief with injections of BoNT-A A compared to placebo injections. A larger sample of patients now needs to be studied in order to identify those who would benefit most from BoNT-A injections for LBP.

## Conclusions

Botulinum toxin injections did not show any efficacy in relieving pain in patients with chronic low-back pain in this randomised controlled trial using a cross-over. Result is in contradiction with the existing literature. With 200UI of Botox injected bilaterally, we did not find any pain relief. But this negative result could also be explained by the lower dose used compared to other studies, and by the low number of patients included. Nevertheless, this negative result could be useful being included in a meta-analysis.

## Abbreviations

BoNT-A: Botulinum Toxin A
LBP: Low-back pain
